# Home-Based HIV Testing for Men Who Have Sex with Men in China: A Novel Community-Based Partnership to Complement Government Programs

**DOI:** 10.1371/journal.pone.0102812

**Published:** 2014-07-22

**Authors:** Jun Tao, Ming-ying Li, Han-Zhu Qian, Li-Juan Wang, Zheng Zhang, Hai-Feng Ding, Ya-Cheng Ji, Dong-liang Li, Dong Xiao, Melissa Hazlitt, Sten H. Vermund, Xiangfei Xiu, Yugang Bao

**Affiliations:** 1 Vanderbilt Institute for Global Health, Vanderbilt University, Nashville, Tennessee, United States of America; 2 Xicheng District Center for Disease Control and Prevention, Beijing, China; 3 Division of Epidemiology, Department of Medicine, Vanderbilt University, Nashville, Tennessee, United States of America; 4 Department of Pediatrics, Vanderbilt University School of Medicine, Nashville, Tennessee, United States of America; 5 Chaoyang District Center for Disease Control and Prevention, Beijing, China; 6 Heibei Medical University, Shijiazhuang, Hebei, China; 7 Chaoyang Chinese AIDS Volunteer Group, Beijing, China; 8 Meharry Medical College, Nashville, Tennessee, United States of America; 9 AHF-China Program, AIDS Healthcare Foundation (AHF), Beijing, China; 10 School of Public Health, Central South University, Changsha, Hunan, China; China Medical University, China

## Abstract

**Background:**

The coverage of HIV testing among Chinese men who have sex with men (MSM) remains low after the scale-up of free HIV testing at government-sponsored testing sites. We evaluated the feasibility of home-based HIV self-testing and the willingness to be HIV tested at community-based organizations (CBO).

**Methods:**

We recruited MSM via on-line advertisement, where they completed an on-line informed consent and subsequent questionnaire survey. Eligible MSM received HIV rapid testing kits by mail, performed the test themselves and reported the result remotely.

**Results:**

Of the 220 men taking a home-based HIV self-testing, 33 MSM (15%) were seropositive. Nearly 65% of the men reported that they were willing to take HIV testing at CBO, while 28% preferred receiving free HIV testing in the government programs at local Centers for Disease Control and Prevention (CDC). Older and lower-income MSM, those who self-reported homosexual orientation, men with no history of sexually transmitted diseases and a lower number of sexual partners in the past six months were associated with preference for taking HIV testing at CBOs. The top three self-reported existing barriers for HIV testing were: no perception of HIV risk (56%), fear of an HIV positive result being reported to the government (41%), and fear of a positive HIV test result (36%).

**Conclusion:**

Home-based HIV self-testing is an alternative approach for increasing the coverage of HIV testing among Chinese MSM. CBO-based HIV testing is a potential alternative, but further studies are needed to evaluate its feasibility.

## Introduction

HIV testing is essential for screening and access to HIV care, including counseling on risk reduction, diagnosis, and initiation of antiretroviral therapy (ART) when indicated [1], [2]. Additionally, early diagnosis and treatment can be expected to reduce transmission to seronegative partners [5]–[7]. Knowledge of HIV infection status is associated with reduction of risky sexual behaviors among persons who test seropositive [3], and early initiation of ART reduced the risk of transmitting HIV among discordant couples by 96% in the HPTN 052 trial [4].

In China, HIV testing is available through two types of government-sponsored programs: HIV voluntary testing and counseling (VCT) clinics and public hospitals. Over 9000 VCT sites have been established mainly in the Centers for Disease Control and Prevention (CDC) at provincial, city or country levels to offer free HIV testing. Alternatively, HIV testing in hospitals is usually provider-initiated and is not free.

Men who have sex with men (MSM) in China have special challenges in accessing both types of HIV testing, including the dual stigma of homosexuality and HIV [8]–[10]. Chinese national HIV/AIDS prevention guidelines suggest that sexually active MSM should be tested at 6 month intervals, but testing frequency remains far below this level. A meta-analysis suggested that average rates of lifetime HIV testing before and after the adoption of China's National Plan for HIV/AIDS Prevention and Control among MSM in 2007 were 24% and 47%, respectively; and the 12-month HIV testing rates were 21% and 38%, respectively, reflecting shortfalls in testing within this community [11]. Frequently cited HIV testing barriers include fear of a positive HIV test result, fear of discrimination by clinic staff for a positive result, and unwillingness to go to a government clinic [12]–[14].

To further expand testing coverage, a complementary approach to VCT and public hospital-based testing is home-based HIV self-testing. HIV test kits are shipped to the user's home, so that they may perform the test in comfort and privacy. Theoretically, home-based HIV self-testing may overcome stated barriers, including lack of time to visit clinics, concern about discrimination, and unwelcome attitudes of clinic staff (perceived or actual). To date, there are few studies to evaluate the feasibility of home-based HIV self-testing among Chinese MSM [15], [16]. We assessed the feasibility and acceptability of home-based HIV self-testing among Chinese MSM, and explored factors associated with seeking HIV testing at CBOs or CDC.

## Methods

### Study procedures and participants in the HIV Home-Based HIV Self-Testing Project

The Home-Based HIV Self-Testing Project was implemented by the Chaoyang Chinese AIDS Volunteer Group (CCAVG) (http://www.hivct.org)—a gay-friendly CBO in Beijing, China, which has actively promoted HIV/AIDS testing and prevention in the MSM community, and has collaborated in conducting HIV/AIDS research projects with MSM for nearly 10 years.

In 2012, CCAVG recruited MSM participants via an advertisement for home-based HIV self-testing posted on its website. When a participant clicked on the link in the advertisement, a new webpage opened for study registration. After reading the project description, they could become study participants by completing an online questionnaire survey, signing the consent, and obtaining a unique ID. The eligibility criteria were: (1) reporting oral or anal sex with a man in the past six months; (2) ≥18 years; (3) HIV negative or unknown status; (4) willingness to complete study procedures.

Eligible men received a HIV rapid test kit (*Human Immunodeficiency Virus HIV ½ Antibody Rapid Test*, ABON Biopharm Co. Ltd, Hangzhou, China) by mail within two business days after they enrolled in the project and made a $10 deposit online for the testing kit. This deposit was refunded to those who completed the test and reported the testing result. To protect personal privacy, the mailed testing kit package was marked as a “gift”.

Participants were offered pre-test counseling via a telephone hotline or QQ Group (Tencent Holdings LLD, www.QQ.com, similar to MSN and Skype). They would then use the HIV rapid test kit for self-testing and read their own test results, with instructions provided by an online video posted to the CCAVG website. Participants were required to take a picture of the testing strip and send it via QQ or email to CCAVG staff, who then provided post-testing counseling and reimbursed the cost of the testing kit. MSM with a negative result were recommended to take a repeat test every six months, and those with a positive result were linked to a local CDC for a second screening test (*Alere Determine HIV-1/2*, Abbott Laboratories, Illinois, USA). For those with dual positives, Western blot (*HIV Blot Version 2.2*, MP Biomedicals Asia Pacific Pte. Ltd, Singapore) was performed to confirm their HIV serostatus. This study was approved by Vanderbilt University School of Medicine (IRB number: 131094).

### Measurement

An online questionnaire was used to collect the following information: (1) socio-demographics including age, ethnicity, marital status, education, occupation, and personal income; (2) sexual behaviors including sexual orientation, anal sexual role, number of male sexual partners and number of unprotected sexual encounters with male partners in the past six months; (3) knowledge, concerns, and preferences for HIV testing, including knowledge about available testing sites, history of HIV testing, fear and concerns about taking HIV tests, and preferences for testing venues; and (4) symptoms of sexually transmitted diseases (STDs).

### Data analysis

We described the demographic and sexual behavioral characteristics by HIV status and performed logistic regression analysis on the association of demographic and sexual behavioral factors with willingness to take HIV testing at CBOs and CDC. We established confounders for each covariate based on *a priori* and directed acyclic graphs (DAG), constructing multivariate logistic regression models for assessing the factors associated with the willingness to be HIV tested at a CBO or CDC. We conducted all the analyses using *Statistical Analysis System* (SAS 9.3, SAS Institute Inc., Cary, NC, USA).

## Results

### Socio-demographic and behavioral characteristics

Of 220 participants, the median age was 26 years (interquartile range [IQR]: 23–30); the majority was ethnically Han (92.7%), single (81.8%), employed (76.4%), and college educated (86.8%). Over a quarter (29.5%) had a monthly personal income greater than 5,000 Chinese yuan (≈US$ 800).

Eighty percent of participants reported to have a homosexual orientation. Nearly a quarter (23.2%) preferred an insertive role when having anal sex with men, 30.9% preferred a receptive role, and 45.9% reported to be versatile. The majority had their first sexual encounter with a man in their early twenties. Only 9.1% reported a history of any STD. The median number of male sexual partners in the past six months was 5 (IQR: 3–6).

### Comparison of socio-demographics and sexual behaviors by HIV status

Of 220 participants, 33 (15%) reported a positive HIV self-test result, and all positives were confirmed in subsequent repeat rapid screening and confirmatory Western blot tests. HIV-infected participants were less likely to have college education, and were more likely to have unprotected anal sex and have a larger number of male sexual partners in the past six months than HIV-uninfected participants (Table 1).

**Table 1:** Socio-demographic characteristics and sexual behaviors among Chinese men who have sex with men who took HIV home testing.

Demographic or behavior	HIV testing result		Total	*P* value†
	Positive (N = 33)	Negative (N = 187)		
Age (median, IQR)	28 (22, 29)	26 (24, 30)	26 (23, 30)	0.84‡
Ethnicity				0.77
Han majority	31 (93.9)	173 (92.5)	204 (92.7)	
Other minorities	2 (6.1)	14 (7.5)	16 (7.3)	
Marital status				0.33
Single	29 (87.9)	151 (80.7)	180 (81.8)	
Currently or ever married	4 (12.2)	36 (19.3)	40 (18.2)	
Education				**0.003**
College or above	23 (69.7)	168 (89.8)	191 (86.8)	
High school or under	10 (30.3)	19 (10.2)	29 (13.2)	
Occupation				0.22
Employed	28 (84.8)	140 (74.9)	168 (76.4)	
Unemployed	5 (15.2)	47 (25.1)	52 (23.6)	
Personal salary per month (Chinese Yuan)				0.92
<5000	23 (69.7)	132 (70.6)	155 (70.5)	
≥5000	10 (30.3)	55 (29.4)	65 (29.5)	
Sexual orientation				0.45
Homosexual	28 (84.8)	148 (79.1)	176 (80.0)	
Bisexual	5 (15.2)	39 (20.9)	44 (20.0)	
Preferred anal sex position				0.30
Insertive	6 (18.2)	45 (24.1)	51 (23.2)	
Receptive	14 (42.4)	54 (28.8)	68 (30.9)	
Dual	13 (39.4)	88 (47.1)	101 (45.9)	
Ever took HIV test before				0.50
No	15 (45.5)	97 (51.9)	112 (50.9)	
Yes	18 (54.5)	90 (48.1)	108 (49.1)	
Self-reported history of sexually transmitted disease				0.52
No	31 (93.9)	169 (90.4)	200 (90.9)	
Yes	2 (6.1)	18 (9.6)	20 (9.1)	
Unprotected anal sex in the past six months§				**0.02**
No	14 (45.2)	100 (67.1)	114 (63.3)	
Yes	17(54.8)	49 (32.9)	66 (36.7)	
No. of male sexual partners in the past six months (median, IQR)				**0.03** ‡
	5 (3, 6)	5 (3,6)	220	

**Note:** Numbers in parentheses are percentages. IQR-interquartile range.

† Chi-square test;

‡ Satterthwaite approximation t-test;

§ Smaller sample size due to missing data.

### Barriers of HIV testing

Nearly half (49%, 108/220) of the participants had ever tested for HIV before; of whom, 61.1% had tested in the past 12 months. Among the 33 HIV-positive participants, 54.5% had ever been previously tested. There was no difference in the testing history along HIV status.

**Figure 1:**
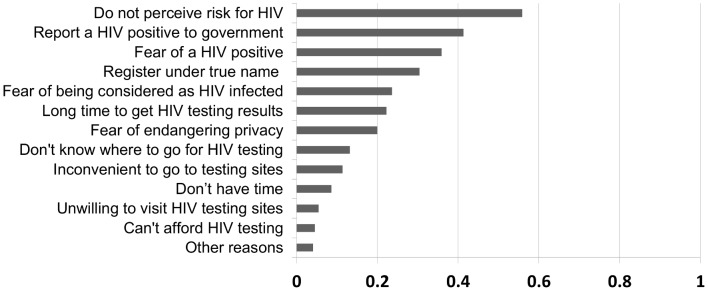
Self-reported barriers for HIV testing among 220 MSM those who took HIV home testing in this study.

**Figure 2:**
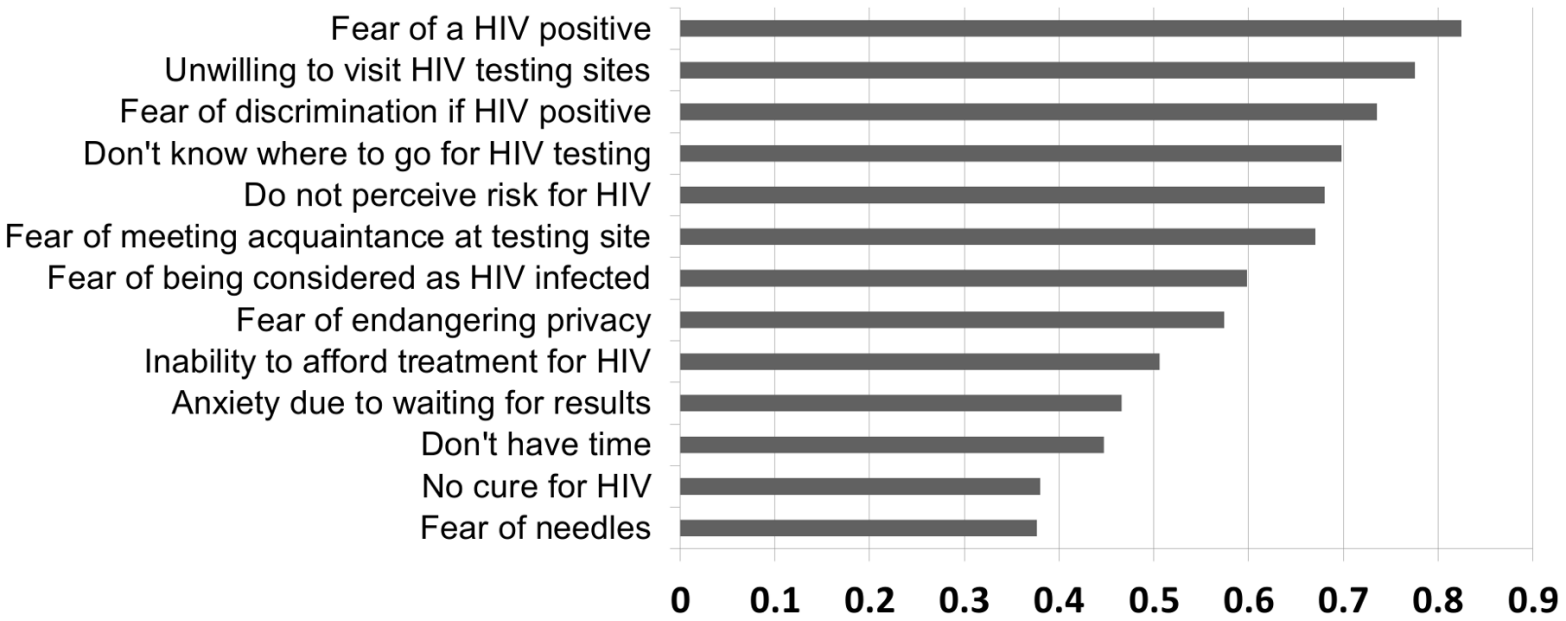
Self-reported barriers for HIV testing among 495 MSM who took clinic-based testing in a contemporaneous parallel study (Li X, *et al* [9]).

In our study sample, the top five barriers for taking a HIV test were: no perception of HIV risk (55.9%), fear of a HIV positive result being reported to the government (41.4%), fear of a HIV positive result (35.9%), reluctance to register for a test under their true names (30.5%), and fear of being considered as HIV-infected whatever the test outcome (23.6%) (Figure 1). In comparison, in a community-based epidemiological study in Beijing City of 495 MSM participants, unwillingness to go to designated HIV testing sites and not knowing where to get HIV testing were among the top five barriers reported (Figure 2).

### Willingness to take HIV testing at CBO

Of 220 participants, 143 (65.0%) reported that they were willing to take HIV testing at a CBO, with Table 2 describing factors associated with this willingness. Men were less apt to be HIV tested at a CBO if they had higher income (adjusted OR [aOR], 0.53; 95% CI: 0.27–1.03), bisexual orientation (OR, 0.46; 95% CI, 0.23–0.89), or a history of STD (aOR, 0.14; 95% CI, 0.05–0.44). For every increase in number of sexual partners by one in the past six months, there was an associated 16% less willingness to be HIV tested at a CBO (aOR, 0.84; 95% CI, 0.75–0.93).

**Table 2:** Factors associated with willingness to go to community-based organization (CBO) for taking HIV testing among Chinese men who have sex with men.

Factors	Willingness to take HIV testing in CBO		Crude OR (95% CI)	Adjusted OR (95% CI)
	Yes (%)	No (%)		
Age (every 1-year increase)*	26 (23, 30)	26 (23, 29)	1.05 (0.99, 1.11)	
Ethnicity				
Han	135 (94.4)	69 (89.6)	1	
Minorities	8 (5.6)	8 (10.4)	0.51 (0.18, 1.42)	
Education				
College or above	123 (86.0)	68 (88.3)	1	
High school or under	20 (14.0)	9 (11.7)	1.23 (0.53, 2.85)	1.13 (0.48, 2.66)†
Marital status				
Single	115 (80.4)	65 (84.4)	1	
Currently or ever married	28 (19.6)	12 (15.6)	1.32 (0.63, 2.77)	0.90 (0.36, 2.25)‡
Salary/per month				
<5000 yuan	105 (73.4)	50 (64.9)	1	
≥5000 yuan	38 (26.6)	27 (35.1)	0.67 (0.37, 1.22)	0.53 (0.27, 1.03)‡
Sexual orientation				
Homosexual	121 (84.6)	55 (71.4)	1	
Bisexual	22 (15.4)	22 (28.6)	0.46 (0.23, 0.89)	
Preferred anal sex position				
Insertive	38 (26.6)	13 (16.9)	1	
versatile	65 (45.4)	36 (46.7)	0.62 (0.29, 1.31)	
Receptive	40 (28.0)	28 (36.4)	0.49 (0.22, 1.08)	
Ever took HIV testing before				
No	65 (45.4)	47 (61.0)	1	
Yes	78 (54.6)	30 (39.0)	1.88 (1.07, 3.30)	1.32 (0.70, 2.53)§
Self-report history of sexually transmitted disease				
No	138 (96.5)	62 (80.5)	1	
Yes	5 (3.5)	15 (19.5)	0.15 (0.05, 0.43)	0.14 (0.05, 0.44)¶
Unprotected anal sex in the past six months#				
No	82 (63.6)	32 (62.8)	1	
Yes	47 (36.4)	19 (37.2)	0.97 (0.49, 1.89)	1.23 (0.56, 2.69)¶
No. of male sexual partners in the past six months* #				
	3 (1,5)	3 (1,7)	0.87 (0.78, 0.96)	0.84 (0.75, 0.93)**

**Note:** Numbers in parentheses are percentages. OR: odds ratio; CI: confidence interval; CBO: community-based organization.

*covariates presented by median and interquartile range;

† Adjusted for age and ethnicity;

‡ Adjusted for age, ethnicity and education;

§ Adjusted for age, ethnicity, education, sexual orientation, sexual role and self-reported STDs history;

¶ Adjusted for age, ethnicity, education, sexual orientation, and sexual role;

# Sample size is smaller due to missing data;

**Adjusted for age, ethnicity, and sexual orientation.

### Willingness to take HIV testing at CDC

Only 61 (27.7%) participants reported willingness to take free HIV testing at a CDC and only one factor was independently associated with this willingness. Contrary to willingness to be tested at a CBO, for every increase in number of sexual partners by one in the past six months, there was an associated 20% increase in likelihood to be HIV tested at a CDC (aOR: 1.20, 95% CI: 1.08, 1.34). Higher income had a marginally significant association (aOR: 1.40, 95% CI: 0.99, 1.98)(Table 3).

**Table 3:** Factors associated with willingness to go to local CDC for taking HIV testing among Chinese men who have sex with men.

Factors	Willingness to take HIV testing in CDC		Crude Odds Ratio (95% CI)	Adjusted OR (95% CI)
	Yes (%)	No (%)		
Age (every 1-year increase)*	26 (24, 29)	26 (23, 30)	0.98 (0.93, 1.04)	
Ethnicity				
Han	55 (90.2)	149 (93.7)	1	
Minorities	6 (9.8)	10 (6.3)	1.62 (0.46, 5.20)	
Education				
College or above	53 (86.9)	138 (86.8)	1	
High school or under	8 (13.1)	21 (13.2)	0.99 (0.36, 2.51)	1.05 (0.44, 2.54)†
Marital status				
Never married	53 (86.9)	137 (79.9)	1	
Current/ever married	8 (13.1)	32 (20.1)	0.60 (0.22, 1.44)	0.55 (0.20, 1.51)‡
Salary/per month				
<5000 yuan	38 (62.3)	117 (73.6)	1	
≥5000 yuan	23 (37.7)	42 (26.4)	1.68 (0.85, 3.29)	1.40 (0.99, 1.98)‡
Sexual orientation				
Homosexual	49 (80.3)	127 (79.9)	1	
Bisexual	12 (19.7)	32 (20.1)	0.97 (0.42, 2.13)	
Preferred anal sex position				
Insertive	15 (24.6)	36 (22.6)	1	
Dual/versatile role	23 (37.7)	78 (49.1)	0.71 (0.33, 1.52)	
Receptive	23 (37.7)	45 (28.3)	1.23 (0.56, 2.69)	
Ever took HIV testing before				
No	27 (44.3)	85 (53.5)	1	
Yes	34 (55.7)	74 (46.5)	1.45 (0.77, 2.74)	1.71 (0.89, 3.28)§
Self-report history of sexually transmitted disease				
No	53 (86.9)	147 (92.5)	1	
Yes	8 (13.1)	12 (7.5)	1.85 (0.62, 5.22)	1.70 (0.62, 4.60)¶
Unprotected anal sex in the past six months#				
No	20 (44.4)	46 (34.1)	1	
Yes	25 (55.6)	89 (65.9)	1.55 (0.73, 3.25)	1.29 (0.61, 2.74)¶
No. of male sexual partners in the past six months* #				
	3 (2,7)	2 (1, 5)	1.19 (1.07, 1.33)	1.20 (1.08, 1.34)**

**Note:** Numbers in parentheses are percentages. OR: odds ratio; CI: confidence interval; CBO: community-based organization.

*covariates presented by mean and interquartile range;

† Adjusted for age and ethnicity;

‡ Adjusted for age, ethnicity and education;

§ Adjusted for age, ethnicity, education, sexual orientation, sexual role and self-reported STDs history;

¶ Adjusted for age, ethnicity, education, sexual orientation, and sexual role;

# Sample size is smaller due to missing data;

**Adjusted for age, ethnicity, and sexual orientation.

## Discussion

The MSM participants in this home-based HIV self-testing project were recruited through an Internet advertisement, and they represent a subgroup of MSM with unique demographics: they tended to be younger (in their twenties), single, and college educated. The distinct demographic profile differences of MSM recruited via the Internet vs. conventional venues were also noted in a previous epidemiologic study in China [17]. It was suggested that young and well-educated MSM are more willing to use Internet-based HIV testing service and interventions. All 33 self-testing positive MSM in our study were linked to a local CDC for HIV confirmatory testing. This suggested that home-based HIV self-testing with linkage-to-care is acceptable and feasible among young and well-educated MSM.

Home-based HIV self-testing can offer an alternative HIV testing venue for MSM who have concerns about HIV testing in conventional venues like a CDC or hospital clinics. This strategy has multiple appeals to MSM, such as reduced concerns about confidentiality and privacy, more flexibility in taking the test, and elimination of long waiting periods for screening results [18]. However, there are also multiple concerns regarding home-based HIV self-testing, including the individual-dependent ability to correctly perform the test, inability to deliver adequate pre-and post-test counseling, and potential gap in linking those with a positive self-screening test to care [19], [20]. Our model of home-based HIV self-testing with linkage-to-care by a gay-friendly CBO innovatively addressed these concerns. Online video instruction was proved to be helpful in guiding men on correct performance of the test and reading the result. The experienced staff at CCAVG were also available to provide pre-test and post-test counseling via hotline and QQ. All participants with a positive self-testing result were successfully tracked through an incentivized mechanism of reimbursing the cost of testing kits, and were linked to a local CDC for confirmatory testing and HIV care. Therefore, this model can play a complementary role in HIV testing, prevention, and treatment among MSM, especially for those who do not initiate HIV testing and care in traditional government-sponsored venues in China.

Self-reported attitudes also suggested another complementary HIV testing venue for gay CBO-based HIV testing [21]. Nearly two thirds (65.0%) of participants were willing to be tested at a CBO, while only 27.7% were willing to go to a CDC. Men were more willing to take a test at a CBO, if they had lower income and self-reported homosexual orientation and did not have a history of STD. Gay-friendly CBOs have multiple advantages in providing HIV testing for MSM. Peer counselors and staff at these CBOs may be more likely to have empathetic attitudes to their clients. They may also have experiences with the same challenges that their clients face. In terms of other marginalized groups, studies among people who injected drugs also showed that they felt less stigmatized at CBOs and were more comfortable to receive services from CBOs than from government clinics [22], [23]. These positive relationships could be further expanded, including the potential for peer counselors and staff to accompany clients taking confirmatory HIV testing at government clinics, so that immediate linkage of HIV-infected MSM to risk reduction and ART could ensue. Therefore, CBO-based HIV testing can contribute to increasing the coverage of HIV testing and could potentially improve the self-empowerment and quality of life of HIV-infected MSM through respectful, community based channels. While the majority of individuals regardless of sexual orientation seek HIV testing at the over 9,000 government-sponsored HIV voluntary counseling and testing sites across the nation, both gay-friendly CBO-based HIV testing and home-based self testing can be supplementary (Figure 2).

Although 87% of participants knew where to go for HIV testing (which was much higher than that in previous studies, [12], [14], [24]), only 49% had been HIV tested before. The perceived barriers for HIV testing in our study sample included low/no perception of HIV risk and fear of a HIV positive result, but the percentage of reporting these barriers was much less than that among MSM participants in a community-based epidemiological study (Figure 2) [14]. The reasons why our study participants had more testing knowledge and fewer barriers might be their younger age and higher educational level. Since many of our participants had substantial concerns about registering their true names or reporting their HIV status to the government, home-based HIV testing offers a viable strategy to overcome these barriers.

**Figure 3:**
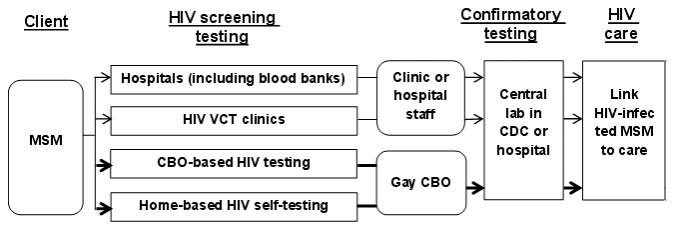
Linking HIV home testing MSM into public health programs among Chinese MSM through gay community based organization (CBO). CDC: center for disease control and prevention; VCT: voluntary counseling and testing.

While our study proposed an innovative model of HIV testing for MSM in China (Figure 3), it also has limitations. We only evaluated the outcomes among participants from Beijing City where the gay CBO CCAVG could link those with a positive self-screening test to confirmatory testing service in the same city. Considering the migration of the MSM population, further research is needed to address the feasibility of expanding home self-testing service to rural and remote areas, and the logistic issues of delivering testing kits. In addition, our study participants were relatively young and well educated Internet-users, and the acceptance of home self-testing among our study participants may not be generalizable to all MSM. Further research is needed to explore the strategy for increasing HIV testing among non-Internet users.

In conclusion, our study demonstrated the feasibility of home-based HIV self-testing among young and well-educated MSM who have access to the Internet, but are nonetheless at considerable risk of HIV infection (fully 15% tested positive for the first time). Home-based HIV self-testing is feasible and complementary to government-sponsored HIV testing programs and the benefits of gay-friendly CBO-based testing seems highly promising. Further research is needed to determine the potential scalability of this approach. Non-invasive HIV testing (oral fluid HIV rapid testing) and conventional HIV testing with internet-facilitated recruitment should be also considered as potential alternative approaches [15], [25], [26]. Multiple options for facility-based and home-based HIV testing might be necessary to maximize the HIV testing coverage among Chinese MSM.
